# Maximising response rates in household telephone surveys

**DOI:** 10.1186/1471-2288-8-71

**Published:** 2008-11-03

**Authors:** Joanne O'Toole, Martha Sinclair, Karin Leder

**Affiliations:** 1 Department of Epidemiology and Preventive Medicine, Monash University, The Alfred, Melbourne, Victoria 3004, Australia; 2 CRC for Water Quality and Treatment, Salisbury, 5108, South Australia

## Abstract

**Background:**

Epidemiological and other studies that require participants to respond by completing a questionnaire face the growing threat of non-response. Response rates to household telephone surveys are diminishing because of changes in telecommunications, marketing and culture. Accordingly, updated information is required about the rate of telephone listing in directories and optimal strategies to maximise survey participation.

**Methods:**

A total of 3426 households in Sydney, Australia were approached to participate in a computer assisted telephone interview (CATI) regarding their domestic (recycled and/or drinking) water usage. Only randomly selected households in the suburb and postcode of interest with a telephone number listed in the Electronic White Pages (EWP) that matched Australian electoral records were approached.

**Results:**

The CATI response rate for eligible households contacted by telephone was 39%. The rate of matching of electoral and EWP records, a measure of telephone directory coverage, was 55%.

**Conclusion:**

The use of a combination of approaches, such as an advance letter, interviewer training, establishment of researcher credentials, increasing call attempts and targeted call times, remains a good strategy to maximise telephone response rates. However, by way of preparation for future technological changes, reduced telephone number listings and people's increasing resistance to unwanted phone calls, alternatives to telephone surveys, such as internet-based approaches, should be investigated.

## Background

Epidemiological and other studies that require participants to respond by completing a questionnaire face the growing threat of non-response. Non-response, an important component of selection bias, is dependent upon the recruitment strategy employed. In the study reported here, telephone directory listings and a telephone questionnaire were used to randomly recruit households from a housing development in Australia to determine their exposure to recycled water, thereby providing information for input into national recycled water health guidelines.

A number of strategies reported as being successful in the literature were employed to maximise telephone response rates:

i. Despatch of a personally addressed introductory letter in advance of a telephone approach [1-4]

ii. Increasing the number of call attempts [3]

iii. Targeting call times [3]

iv. Training of interviewers [3]

v. Establishment of credentials and survey significance at the start of the interview [5].

This paper reports on the combined success of these strategies to maximise telephone response rates in the context of telecommunication changes and on the scope of the problem of using a telephone database for recruitment of a representative Australian household sample.

## Methods

This study was conducted as a University survey and was approved by Monash University Standing Committee on Ethics in Research involving Humans. Target households were located in a housing development (total 16,000 households) in metropolitan Sydney supplied with conventional tap water and recycled water through separate pipe systems. Households located within metropolitan Sydney, supplied with drinking water only were used as controls. A computer assisted telephone interview (CATI) of approximately 15 minutes duration was administered to householders from February to April 2006 immediately following the conduct of a pilot study (November 2005 to January 2006) performed to assess the logistical requirements and likely reduction in numbers at each step of household selection and recruitment.

To obtain the names and addresses of householders, electoral information was acquired. In Australia, voting is compulsory and the electoral roll provides an easily accessible and up to date means of contacting persons for health research studies. Records were grouped according to electorate, suburb, postcode, street number and address resulting in a list of households (single dwellings) located in the areas of interest. Random sequences of target and control households were generated, and for the first 3500 households in each group, data matching with the Electronic White Pages (EWP) was performed for the purpose of obtaining telephone numbers. The generated random sequence of households was the order in which data matching with EWP was performed and introductory letters were sent. Telephone matching of records was only performed for 7000 households (3500 households from each group) as pilot study results indicated that this would be sufficient to achieve a total sample size of 1000 CATIs. The sample size of 500 target and 500 control households for the main study was based both on budgetary considerations and on a required minimum of 200 target households also completing and returning a water activity diary (allowing for a comparison of diary and CATI responses corresponding to a 95% confidence interval of 0.5–0.7 where the weighted kappa statistic is 0.6 [6]) (diary results are not reported here).

Elector households with a listed telephone number in the EWP were sent an introductory letter inviting them to participate in the study. Telephone contact was commenced one to three weeks after the introductory letter despatch. Four telephone contact attempts were made before contact was terminated. The majority of telephone calls were made between 6 pm and 9 pm based on pilot study results showing that highest contact rates with householders were obtained within this time period. Interviewers were trained in the administration of the CATI and were provided with a preamble script that reinforced both the credentials of the University and the health related outcome of the study.

The CATI response rate was computed as the number of completed interviews divided by the number of eligible and/or contacted households. Eligibility for telephone interview comprised persons of 18 years of age or older, residing in single housing units within suburbs of interest with sufficient command of English to complete the CATI. For the target households an additional eligibility criterion was supply of recycled water (not all households in target suburbs are supplied with recycled water for geographical and/or historical reasons). Three additional versions of the response rate were also calculated. In the first, the total number of households to which letters were sent was used as the denominator. This represents a theoretical lower limit to the response rate, as it includes both eligible and ineligible households. In the second version, an estimate of the total number of eligible households to which letters were sent was used as the denominator; this estimate was arrived at by assuming that the rate of eligibility among households not contacted was the same as that among households that were contacted. In the third version, the total number of households contacted was used as the denominator.

## Results

Table 1 shows household recruitment results. These results show that the rate of matching of electoral and EWP records was 55% (3864 out of 7000 households). The average CATI response (completion) rate for 2663 eligible households (1262 target and 1401 control households) contacted by telephone was 39%.

**Table 1:** Household recruitment results for recycled water exposure study

**Parameter**	**Target (recycled water) households**	**Control (drinking water only) households**
Total number of households	16,000 (approx)	Unlimited

Households in the delimited AEC sampling frame	13,940	10,097

Households randomly selected from delimited electoral sampling frame for data matching with EWP database	3500	3500

Households with listed EWP telephone number	1995	1869

Number of introductory letters sent (pilot study)	295	143

Number of introductory letters sent*	1700	1726

Number of households successfully contacted by telephone*	1410	1444

Number of eligible^# ^households successfully contacted by telephone*	1262	1401

Households with an invalid telephone number*	72	66

Number of completed interviews*	523	514

AEC = Australian Electoral Commission EWP = Electronic White pages

* = main study

^# ^Eligibility = persons of 18 years of age or older, residing in single housing units within suburbs of interest with sufficient command of English. For the target households an additional eligibility criterion was supply of recycled water

Overall 84% of households were contacted by telephone and only 4% of households had an invalid telephone number. The total number of telephone calls made, based on 4 contact attempts before a household was given a 'completed' status, was 7445. After one, two, and three telephone contact attempts, 44.3%, 66.5% and 78.5% of households respectively were able to be given a 'completed' status.

Table 2 shows the CATI response rates using four different denominators for the calculation of results. Response rates (all households) ranged from 30% to 39% depending upon the denominator used for the calculation.

**Table 2:** CATI response rates

**Denominator**	**Response rate (denominator used in calculation)**		
	
	Target households^1^	Control households^2^	All households^3^
Number of letters sent	31% (1700)	30% (1726)	30% (3426)

Number of letters sent to estimated eligible households*	34% (1522)	31% (1674)	33% (3196)

Number of households contacted by telephone	37% (1410)	36% (1444)	36% (2854)

Number of eligible households contacted by telephone	41% (1262)	37% (1401)	39% (2663)

^1 ^523 completed CATI ^2 ^514 completed CATI ^3 ^1037 completed CATI

* = rate of eligibility of households contacted by telephone assumed to be the same for households not contacted by telephone

## Discussion

Results (Table 2) show that even when the CATI response rate is calculated using the number of letters sent as the denominator, a probable underestimate given that some letters would be sent to ineligible households, it is higher at 30% (all households) than that obtained for similar water usage studies conducted by Sydney Water Corporation (SWC) in the same period (Sydney Water Corporation, personal communication). In a 2005 residential survey performed by SWC where response rates were calculated using the number of eligible households contacted by telephone as the denominator, 1600 households (or 18%) responded fully out of 9729 potential contacts drawn from the EWP, of which 778 were ineligible. This compares with a response rate of 39% (all households) obtained for this study when response rates were calculated in exactly the same way. In another SWC study, where the internal client database was used and letters were sent to householders offering them an incentive to undertake interviews in their home, the response rate was 24%. In this latter case, householders were required to contact the water authority by telephone if they wished to participate in the survey (Sydney Water Corporation, personal communication). Taken together these comparisons show that irrespective of the way in which the CATI response rate is calculated, response rates in this study are between 25% – 116% higher than those obtained for water authority initiated surveys. This suggests that the combination of strategies used in this study to maximise telephone survey response rates was effective and that these strategies should continue to be used.

A limitation of the study is that the effectiveness of each of the individual strategies employed could not be assessed. It is therefore possible that some of the strategies may have prevented the response rate from being even higher. We regard this as unlikely based on documented enhanced telephone response rates of a personally addressed introductory letter in advance of a telephone approach [1-4] and the short time (less than 3 weeks) between the letter and telephone contact. In addition, all comments about the letter if made by the householder on contact were positive. Also, contact rates increased progressively with the number of call attempts and the final telephone contact rate was high at 84%. Furthermore, results of the pilot study showed that rate of telephone contact was highest for the 6–9 pm time frame when the majority of telephone contact attempts were made. Despite recent lobbying for, and support of, "Do not call" registers in Australia, giving a strong indication of attitudes to unwanted phone calls, the high response rate relative to water authority studies also suggests that the establishment of University credentials and significance of the survey at the start of the telephone interview assisted to maximise response rates. Training of interviewers and establishment of credentials and survey significance are also practices documented as improving response rates [5].

The high telephone contact rate (84%) and low rate of invalid telephone numbers (4%) validate the accuracy of the EWP. However, it is the decreasing coverage of the EWP that is of concern for future studies. Although it is difficult to obtain information from telecommunication service providers about the rate of telephone listing in directories, it is important that such rates are monitored so that recruitment strategies can be adjusted accordingly. The 45% of households without a listed telephone number in this study compares with a reported estimate of 13% in 1996 [7], demonstrating the impact of changing patterns of telephone ownership and the increasing availability of new technology.

When the percentage of target households with listed telephone numbers (57%) is combined with an estimated coverage rate of the filtered Australian Electoral Commission (AEC) database (13,940 out of 16,000 target households), the overall coverage of the combined AEC and EWP sampling frames for this study approximates at 50%. Despite this selection of only one half of the target households using the combined AEC and EWP sampling frame, we believe the strategy employed nonetheless maximised the number of completed CATIs compared with alternatives. In the absence of name and address details it is not possible to use the EWP to locate target households nor is there an ability to send a primer letter about the study in advance of a telephone approach. AEC records and client databases are alternative means of obtaining name and address information. However, use of water authority client records by independent researchers is provisional upon privacy provisions to householders being met and ethics approval being given for the water authority to provide this information. Random digit dialling, another possible alternative to select households for telephone surveys does not permit the sending of a primer letter and household selection is problematic. For example, the 16,000 target households in this study were covered by four telephone exchanges and at least 11 non-sequential sets of telephone numbers. In addition, use of telephone numbers for other telecommunication options (e.g. internet or fax) makes it more difficult to select a household from a random digit sampling frame [8]. The use of AEC records alone to administer a postal survey was not contemplated because of the number of survey questions and low participation rates of postal surveys.

The ability to select an average of 55% of households for telephone interview based on their listing in the EWP is a limitation of the selection and recruitment strategy employed leading to questions about the representativeness of the sample households. While it is possible that the water-using behaviour of listed and non-listed households in the EWP does vary, we consider that EWP listing is unlikely to be a primary determinant in the volume of water used by households. The relative uniformity in household characteristics that drive water usage such as garden area, garden age and household size in the entire target survey area independent of EWP listing supports this assumption. However, water usage by households within each of the target and control areas may not be homogenous. Those households that completed the CATI may have different water usage to those refusing to complete the CATI based on a greater commitment to water recycling schemes or water conservation. This bias is unavoidable and applies to all surveys whether administered by post, personal interview or the Internet.

A possible emerging alternative to telephone surveys is the use of the Internet. However, despite increasing access of the Australian population to the Internet at home (60% in 2006) [9], a relatively small proportion of the population have registered in on-line databases. Such databases thus currently provide a smaller available sample for survey purposes than do telephone databases. The utility of Internet databases to produce a large enough sample is further eroded when 'niche' households in a small number of suburbs are to be selected as in this study. It is not until the diminishment in telephone directory listings, coupled with increasing resistance to unwanted phone calls, reaches a point of 'cross over' with an increase in Internet access and database registration, that on-line surveys, rather than telephone surveys, will become the preferred survey method. In the interim period prior to this 'cross over' it is important that telephone surveys are supported by good strategies to maximise survey participation. Continued monitoring of both the rate of household listings in telephone directories and registration in on-line databases is also required so this point of 'cross over' is known.

## Conclusion

The use of a combination of approaches, such as an advance letter, interviewer training, establishment of researcher credentials, increasing call attempts and targeted call times, remains a good strategy to maximise telephone response rates. Household telephone number listings in directories are decreasing and it is important that listing rates are monitored to inform future survey design. By way of preparation for future technological changes, reduced telephone number listings and people's increasing resistance to unwanted phone calls, alternatives to telephone surveys, such as Internet-based approaches, should be investigated.

## Competing interests

The authors declare that they have no competing interests in relation to this work. All authors had full access to data in the study and had final responsibility for the decision to submit for publication.

## Authors' contributions

JO was responsible for the conduct of the telephone survey, design of the paper and analysis and interpretation of data.

MS was involved in the design concept of the project and reviewed the final manuscript for intellectual content.

KL was involved in the design concept of the project, design, drafting and revision of the final manuscript for intellectual content.

## Pre-publication history

The pre-publication history for this paper can be accessed here:


